# Bioterrorism as a public health threat.

**DOI:** 10.3201/eid0403.980340

**Published:** 1998

**Authors:** D A Henderson

**Affiliations:** Johns Hopkins University, Baltimore, Maryland, USA. dahzero@aol.com

## Abstract

The threat of bioterrorism, long ignored and denied, has heightened over the past few years. Recent events in Iraq, Japan, and Russia cast an ominous shadow. Two candidate agents are of special concern--smallpox and anthrax. The magnitude of the problems and the gravity of the scenarios associated with release of these organisms have been vividly portrayed by two epidemics of smallpox in Europe during the 1970s and by an accidental release of aerosolized anthrax from a Russian bioweapons facility in 1979. Efforts in the United States to deal with possible incidents involving bioweapons in the civilian sector have only recently begun and have made only limited progress. Only with substantial additional resources at the federal, state, and local levels can a credible and meaningful response be mounted. For longer-term solutions, the medical community must educate both the public and policy makers about bioterrorism and build a global consensus condemning its use.


### Bioterrorism as a Public Health Threat

D.A. Henderson
The Johns Hopkins University, Baltimore, Maryland, USA

The threat of bioterrorism, long ignored and denied, has heightened over the past few years. Recent events in Iraq, Japan, and Russia cast an ominous shadow. Two candidate agents are of special concern—smallpox and anthrax. The magnitude of the problems and the gravity of the scenarios associated with release of these organisms have been vividly portrayed by two epidemics of smallpox in Europe during the 1970s and by an accidental release of aerosolized anthrax from a Russian bioweapons facility in 1979. Efforts in the United States to deal with possible incidents involving bioweapons in the civilian sector have only recently begun and have made only limited progress. Only with substantial additional resources at the federal, state, and local levels can a credible and meaningful response be mounted. For longer-term solutions, the medical community must educate both the public and policy makers about bioterrorism and build a global consensus condemning its use.

Until recently, biological terrorism had been little discussed or written about. Until recently, I had doubts about publicizing the subject because of concern that it might entice some to undertake dangerous, perhaps catastrophic experiments. However, events of the past 12 to 18 months have made it clear that likely perpetrators already envisage every possible scenario.

Four points of view prevalent among national policy circles and the academic community at various times have served to dismiss biological terrorism as nothing more than a theoretical possibility. l) Biological weapons have so seldom been deployed that precedent would suggest they will not be used. 2) Their use is so morally repugnant that no one would deign to use them. 3) The science of producing enough organisms and dispersing them is so difficult that it is within the reach of only the most sophisticated laboratories. 4) Like the concept of a "nuclear winter," the potential destructiveness of bioweapons is essentially unthinkable and so to be dismissed. Each of these arguments is without validity.

Nations and dissident groups exist that have both the motivation and access to skills to selectively cultivate some of the most dangerous pathogens and to deploy them as agents in acts of terrorism or war. After the Gulf War, Iraq was discovered to have a large biological weapons program. In 1995, Iraq confirmed that it had produced, filled, and deployed bombs, rockets, and aircraft spray tanks containing *Bacillus anthracis* and botulinum toxin (1,2); its work force and technologic infrastructure are still wholly intact. Also in 1995, the Japanese cult, Aum Shinrikyo, released the nerve gas Sarin in the Tokyo subway. The cult also had plans for biological terrorism (3); included in its arsenal were large quantities of nutrient media, botulinum toxin, anthrax cultures, and drone aircraft equipped with spray tanks. Members of this group had traveled to Zaire in 1992 to obtain samples of Ebola virus for weapons development.

Of more recent concern is the status of one of Russia's largest and most sophisticated former bioweapons facilities, called Vector, in Koltsovo, Novosibirsk. Through the early 1990s, this was a 4,000-person, 30-building facility with ample biosafety level 4 laboratory facilities, used for the isolation of both specimens and human cases. Situated on an open plain surrounded by electric fences and protected by an elite guard, the facility housed the smallpox virus as well as work on Ebola, Marburg, and the hemorrhagic fever viruses (e.g., Machupo and Crimean-Congo). A visit in the autumn of 1997 found a half-empty facility protected by a handful of guards who had not been paid for months (P. Jahrling, pers. comm., 1998). No one can say where the scientists have gone, nor is there confidence now that this is

Address for correspondence: D.A. Henderson, The Johns Hopkins University, The Johns Hopkins School of Medicine, 615 North Wolfe Street, Baltimore, MD 21205, USA; fax: 410-955-6898; e-mail: dahzero@aol.com.

the only storage site for smallpox virus outside the Centers for Disease Control and Prevention.

The number of countries engaged in biological weapons experimentation has grown from 4 in the 1960s to 11 in the 1990s (4). Meanwhile, the bombing of the World Trade Center and the Oklahoma City Federal Building have dramatized the serious problems even small dissident groups can cause.

A comprehensive review of the problems posed by biological terrorism and warfare has been published (5). Four observations deserve special note. First, biological terrorism is more likely than ever before and far more threatening than either explosives or chemicals. Second, official actions directed at the threat to the civilian population (less than 2 years in the making) have been only marginally funded and minimally supported (6). Third, preventing or countering bioterrorism will be extremely difficult. Recipes for making biological weapons are now available on the Internet, and even groups with modest finances and basic training in biology and engineering could develop, should they wish, an effective weapon (7) at little cost. Fourth, detection or interdiction of those intending to use biological weapons is next to impossible. Thus, the first evidence of such weapons will almost certainly be cases in hospital emergency rooms. Specialists in infectious diseases thus constitute the front line of defense. The rapidity with which they and emergency room personnel reach a proper diagnosis and the speed with which they apply preventive and therapeutic measures could spell the difference between thousands and perhaps tens of thousands of casualties. Indeed, the survival of physicians and health-care staff caring for the patients may be at stake. However, today few have ever seen so much as a single case of smallpox, plague, or anthrax, or, for that matter, would recall the characteristics of such cases. Few, if any, diagnostic laboratories are prepared to confirm promptly such diagnoses.

Of a long list of potential pathogens, only a handful are reasonably easy to prepare and disperse and can inflict sufficiently severe disease to paralyze a city and perhaps a nation. In April 1994, Anatoliy Vorobyov, a Russian bioweapons expert, presented to a working group of the National Academy of Sciences the conclusions of Russian experts as to the agents most likely to be used (8). Smallpox headed the list followed closely by anthrax and plague. None of these agents has so far effectively been deployed as a biological weapon, and thus no real world events exist to provide likely scenarios. However, we have had several well-documented smallpox importations into Europe over recent decades; two bear recounting.

Smallpox is caused by a virus spread from person to person; infected persons have a characteristic fever and rash. Virus infection invariably results in symptomatic disease. There are no mild, subclinical infections among unvaccinated persons. After an incubation period of 10 to 12 days, the patient has high fever and pain. Then a rash begins with small papules developing into pustules on day 7 to 8 and finally changing to scabs around day 12. Between 25% and 30% of all unvaccinated patients die of the disease. There was, and is, no specific treatment.

Until 1980, essentially all countries conducted vaccination programs of some sort, whether or not they had endemic disease (9). Until 1972, the United States mandated smallpox vaccination for all children at school entry, although the last cases had occurred in 1949, 23 years before. In the United Kingdom, four standby hospitals were to be opened only if smallpox cases were imported, and in Germany, two state-of-the-art isolation hospitals were constructed in the 1960s specifically for the isolation of smallpox cases should they occur.

In 1962, the initial response of U.S. officials to the occurrence of a single case of smallpox illustrated extreme concern. That year, a young Canadian boy returned from Brazil, traveling by air to New York and by train to Toronto by way of Albany and Buffalo (10). Shortly after arrival in Toronto, he developed a rash and was hospitalized. In response to this single case, senior U.S. government officials seriously considered a plan of action that called for the border with Canada to be closed, for mass vaccination campaigns to be conducted in all cities along the route from New York through Albany, Syracuse, Rochester, and Buffalo, and for vaccination of all who had been in Grand Central Station on the day the Canadian boy was there. Sensibly, this plan was soon scrapped for more modest measures, albeit not without considerable debate.

The potential of aerosolized smallpox to spread over a considerable distance and to infect at low doses was vividly demonstrated in an outbreak in Germany in 1970 (11). That year, a German electrician returning from Pakistan became ill with high fever and diarrhea. On January 11, he was admitted to a local hospital and was isolated in a separate room on the ground floor because it was feared he might have typhoid fever. He had contact with only two nurses over the next 3 days. On January 14 a rash developed, and on January 16 the diagnosis of smallpox was confirmed. He was immediately transported to one of Germany's special isolation hospitals, and more than 100,000 persons were promptly vaccinated. The hospital had been closed to visitors because of an influenza outbreak for several days before the patient was admitted. After the diagnosis of smallpox, other hospital patients and staff were quarantined for 4 weeks and were vaccinated; very ill patients received vaccinia-immune globulin first. However, the smallpox patient had had a cough, a symptom seldom seen with smallpox; coughing can produce a large-volume, small-particle aerosol like what might occur after its use as a terrorist weapon. Subsequently, 19 cases occurred in the hospital, including four in other rooms on the patient's floor, eight on the floor above, and nine on the third floor. Two were contact cases. One of the cases was in a visitor who had spent fewer than 15 minutes in the hospital and had only briefly opened a corridor door, easily 30 feet from the patient's room, to ask directions. Three of the patients were nurses, one of whom died. This outbreak occurred in a well-vaccinated population.

An outbreak in Yugoslavia in February 1972 also illustrates the havoc created even by a small number of cases. Yugoslavia's last case of smallpox had occurred in 1927. Nevertheless, Yugoslavia, like most countries, had continued populationwide vaccination to protect against imported cases. In 1972, a pilgrim returning from Mecca became ill with an undiagnosed febrile disease. Friends and relatives visited from a number of different areas; 2 weeks later, 11 of them became ill with high fever and rash. The patients were not aware of each other's illness, and their physicians (few of whom had ever seen a case of smallpox) failed to make a correct diagnosis.

One of the 11 patients was a 30-year-old teacher who quickly became critically ill with the hemorrhagic form, a form not readily diagnosed even by experts. The teacher was first given penicillin at a local clinic, but as he became increasingly ill, he was transferred to a dermatology ward in a city hospital, then to a similar ward in the capital city, and finally to a critical care unit because he was bleeding profusely and in shock. He died before a definitive diagnosis was made. He was buried 2 days before the first case of smallpox was recognized.

The first cases were correctly diagnosed 4 weeks after the first patient became ill, but by then, 150 persons were already infected; of these, 38 (including two physicians, two nurses, and four other hospital staff) were infected by the young teacher. The cases occurred in widely separated areas of the country. By the time of diagnosis, the 150 secondary cases had already begun to expose yet another generation, and, inevitably, questions arose as to how many other yet undetected cases there might be.

Health authorities launched a nationwide vaccination campaign. Mass vaccination clinics were held, and checkpoints along roads were established to examine vaccination certificates. Twenty million persons were vaccinated. Hotels and residential apartments were taken over, cordoned off by the military, and all known contacts of cases were forced into these centers under military guard. Some 10,000 persons spent 2 weeks or more in isolation. Meanwhile, neighboring countries closed their borders. Nine weeks after the first patient became ill, the outbreak stopped. In all, 175 patients contracted smallpox, and 35 died.

What might happen if smallpox were released today in a U.S. city? First, routine vaccination stopped in the United States in 1972. Some travelers, many military recruits, and a handful of laboratory workers were vaccinated over the following 8 years. Overall, however, it is doubtful that more than 10% to 15% of the population today has residual smallpox immunity. If some modest volume of virus were to be released (perhaps by exploding a light bulb containing virus in a Washington subway), the event would almost certainly go unnoticed until the first cases with rash began to appear 9 or 10 days later. With patients seen by different physicians (who almost certainly had never before seen a smallpox case) in different clinics, several days would probably elapse before the diagnosis of smallpox was confirmed and an alarm was sounded.

Even if only 100 persons were infected and required hospitalization, a group of patients many times larger would become ill with fever and rash and receive an uncertain diagnosis. Some would be reported from other cities and other states. Where would all of these patients be admitted? In the Washington, D.C., metropolitan area, no more than 100 hospital beds provide adequate isolation. Who would care for the patients? Few hospital staff have any smallpox immunity. Moreover, one or two patients with severe hemorrhagic cases (which typically have very short incubation periods), who would have been hospitalized before smallpox was suspected, would have been cared for by a large, unprotected intensive care team.

What of contacts? In past outbreaks, contacts of confirmed or suspected cases numbered in the thousands, if not tens of thousands. What measures should or could be taken to deal with such numbers? Would patients be isolated as in Yugoslavia, and if so, where? Logistics could be simplified if rapid, easily used laboratory tests could confirm or rule out smallpox among suspected cases. At present, however, such tests are known only to scientists in two government laboratories.

An immediate clamor for mass vaccination (as in the outbreaks in Germany and Yugoslavia) can be predicted. U.S. stocks of smallpox vaccine are nominally listed at 15 million doses, but with packaging, the useful number of doses is perhaps half that number. How widely and quickly should this vaccine be used? Were vaccine to be limited strictly to close contacts of confirmed cases, comparatively few doses would be needed. However, the realities of dealing with even a small epidemic would almost certainly preclude such a cautious, measured vaccination effort. Vaccine reserves would rapidly disappear, and there is, at present, no manufacturing capacity to produce additional vaccine. If an emergency effort were made to produce new stocks of smallpox vaccine, many months to a year or more would be required.

What of anthrax, which has been so enthusiastically embraced by both Iraq and the Aum Shinrikyo? The organism is easy to produce in large quantity. In its dried form, it is extremely stable. The effect of aerosolized anthrax on humans once had to be inferred from animal experiments and the occasional human infection among workers in factories processing sheep and goat hides (12). It was clear that inhalation of anthrax is highly lethal. Just how lethal became evident in the 1979 Sverdlovsk epidemic (13).

In all, 77 cases were identified with certainty; 66 patients died. The actual total number of cases was probably considerably more than 100. The persons affected lived or worked somewhere within a narrow zone extending some 4 km south and east of a military bioweapons facility. An accidental airborne release of anthrax spores occurred during a single day and may well have lasted no more than minutes. Further investigations revealed anthrax deaths among sheep and cows in six different villages up to 50 km southeast of the military compound along the same axis as the human cases.

Of the 58 patients with known dates of disease onset, only 9 had symptoms within a week after exposure; some became ill as late as 6 weeks after exposure. Whether the onset of illness occurred sooner or later, death almost always followed within 1 to 4 days after onset. However, there appeared to be a somewhat higher proportion of survivors after the fourth week. This almost certainly resulted from the widespread application of penicillin prophylaxis and anthrax vaccine, both of which were distributed in mid-April throughout a population of 59,000.

Meselson and his colleagues, who documented this outbreak, calculate that the weight of spores released as an aerosol could have been as little as a few milligrams or as much as "nearly a gram." Iraq acknowledged producing at least 8,000 L of solution with an anthrax spore and cell count of 109/ml (1). The ramifications of even a modest-sized release of anthrax spores in a city are profound. Emergency rooms would begin seeing a few patients with high fever and some difficulty breathing perhaps 3 to 4 days after exposure. By the time the patients were seen, it is almost certain that it would be too late for antibiotic therapy. All patients would die within 24 to 48 hours. No emergency room physicians or infectious disease specialists have ever seen a case of inhalation anthrax; medical laboratories have had virtually no experience in its diagnosis. Thus, at least 3 to 5 days would elapse before a definitive diagnosis would be made.

Once anthrax was diagnosed, one would be faced with the prospect of what to do over the succeeding 6 to 8 weeks. Should vaccine be administered to those who might have been exposed? At present, little vaccine is available, and no plan exists to produce any for civilian use. Should antibiotics be administered prophylactically? If so, which antibiotics, and what should be the criteria for exposure? What quantity would be required to treat an exposed population of perhaps 500,000 over a 6-week period? Should one be concerned about additional infections resulting from anthrax spores subsequently resuspended and inhaled by others? Should everyone who has been anywhere near the city report to a local physician for treatment at the first occurrence of fever or cough, however mild? Undoubtedly, many would have such symptoms, especially in the winter; how can such symptoms be distinguished from the premonitory symptoms of anthrax that may proceed to death within 24 to 48 hours?

We are ill-prepared to deal with a terrorist attack that employs biological weapons. In countering civilian terrorism, the focus (a modest extension of existing protocols to deal with a hazard materials incident) has been almost wholly on chemical and explosive weapons. A chemical release or a major explosion is far more manageable than the biological challenges posed by smallpox or anthrax. After an explosion or a chemical attack, the worst effects are quickly over, the dimensions of the catastrophe can be defined, the toll of injuries and deaths can be ascertained, and efforts can be directed to stabilization and recovery. Not so following the use of smallpox or anthrax. Day after relentless day, additional cases could be expected, and in new areas.

The specter of biological weapons use is an ugly one, every bit as grim and foreboding as that of a nuclear winter. As was done in response to the nuclear threat, the medical community should educate the public and policy makers about the threat. We need to build on the 1972 Biological and Toxin Weapons Convention to strengthen measures prohibiting the development and production of biological weapons and to ensure compliance with existing agreements. In a broader sense, we need a strong moral consensus condemning biological weapons.

But this is not enough. In the longer term, we need to be as prepared to detect, diagnose, characterize epidemiologically, and respond appropriately to biological weapons use as to the threat of new and reemerging infections. In fact, the needs are convergent. We need at international, state, and local levels a greater capacity for surveillance; a far better network of laboratories and better diagnostic instruments; and a more adequate cadre of trained epidemiologists, clinicians, and researchers.

On the immediate horizon, we cannot delay the development and implementation of strategic plans for coping with civilian bioterrorism. The needed stocking of vaccines and drugs as well as the training and mobilization of health workers, both public and private, at state, city, and local levels will require time. Knowing well what little has been done, I can only say that a mammoth task lies before us.

D.A. Henderson is a distinguished service professor at the Johns Hopkins University, with appointments in the Departments of Health and Epidemiology, School of Hygiene and Public Health. Dr. Henderson directed the World Health Organization's global smallpox eradication campaign (1966-1977) and helped initiate WHO's global program of immunization in 1974. He also served in the federal government as deputy assistant secretary and senior science advisor in the Department of Health and Human Services.

### References

1. Ekeus R. Iraq's biological weapons programme: UNSCOM's experience. Memorandum report to the United Nations Security Council; 1996 20 Nov; New York.
2. Zalinskas RA. Iraq's biological weapons: the past as future? JAMA 1997;278:418-24.
3. Daplan E, Marchell A. The cult at the end of the world. New York: Crown Publishing Group; 1996.
4. Roberts B. New challenges and new policy priorities for the 1990s. In: Biologic weapons: weapons of the future. Washington: Center for Strategic and International Studies; 1993.
5. Bioweapons and bioterrorism. JAMA 1997;278:351-70, 389-436.
6. Tucker JB. National health and medical services response to incidents of chemical and biological terrorism. JAMA 1997;285:362-8.
7. Danzig R, Berkowsky PB. Why should we be concerned about biological warfare? JAMA 1997;285:431-2.
8. Vorobyov A. Criterion rating as a measure of probable use of bio agents as biological weapons. In: Papers presented to the Working Group on Biological Weapons Control of the Committee on International Security and Arms Control, National Academy of Sciences; 1994 Apr; Washington.
9. Fenner F, Henderson DA, Arita I, Jezek Z, Ladnyi I. Smallpox and its eradication. Geneva: World Health Organization; 1988.
10. Epidemiologic report. Smallpox, Canada. MMWR Morb Mortal Wkly Rep 1962;11:258.
11. Wehrle PF, Posch J, Richter KH, Henderson DA. An airborne outbreak of smallpox in a German hospital and its significance with respect to other recent outbreaks in Europe. Bull World Health Organ 1970;4:669-79.
12. Brachman PS, Friedlander AM. Anthrax. In: Plotkin SA, Mortimer EA, editors. Vaccines. Philadelphia: WB Saunders; 1994.
13. Meselson M, Guillemin V, Hugh-Jones M, Langmuir A, Popova I, Shelokov A, et al. The Sverdlovsk anthrax outbreak of 1979. Science 1994;266:1202-8.
